# Role of a Urinary Biomarker in the Common Mechanism of Physical Performance and Cognitive Function

**DOI:** 10.3389/fmed.2022.816822

**Published:** 2022-02-18

**Authors:** Shan Jiang, Ju Cui, Li-Qun Zhang, Zhen Liu, Yan Zhang, Yuan Shi, Jian-Ping Cai

**Affiliations:** ^1^The Key Laboratory of Geriatrics, Beijing Institute of Geriatrics, Beijing Hospital, Beijing, China; ^2^National Center of Gerontology, National Health Commission, Beijing, China; ^3^Institute of Geriatric Medicine, Chinese Academy of Medical Sciences, Beijing, China; ^4^Department of Geriatric Medicine, Longtan Community Health Service Center, Beijing, China

**Keywords:** physical performance, cognitive function, oxidative stress, urinary 8-oxo-7, 8-dihydroguanosine, RNA oxidation

## Abstract

**Introduction:**

Healthy aging is described as a process of developing and maintaining intrinsic abilities, including physical and cognitive functions. Although oxidative stress is a common mechanism shared by loss of muscle strength and dementia, its relationship with decreased physical performance and cognitive impairment remains unclear. We aimed to investigate the role of urinary 8-oxo-7, 8-dihydroguanosine (8-oxoGsn), a biomarker of oxidative damage to RNA, in physical and cognitive decline.

**Methods:**

The study followed a cross-sectional design and recruited 40–94-year-old inhabitants of Beijing, China (471 men and 881 women). The physical performance of the participants was assessed using handgrip strength, walking speed, and the repeated chair stand test. The cognitive function was assessed using the Montreal Cognitive Assessment (MoCA) 5-min protocol. Urinary 8-oxoGsn levels were measured for all participants.

**Results:**

Participants with high urinary 8-oxoGsn levels were more likely to have low grip strength, slow walking speed, poor performance in the repeated chair stand test, and low scores on the MoCA 5-min protocol (odds ratio [OR] 3.43, 95% confidence interval [CI]: 1.52–7.76; OR 1.71, 95% CI: 1.16–2.53; OR 2.06, 95% CI: 0.92–4.63; OR 1.75, 95% CI: 1.18–2.58), after adjusting for age, sex, smoking habits, alcohol consumption, hypertension, diabetes, cerebro-cardiovascular disease, and chronic kidney disease.

**Conclusion:**

Elevated levels of oxidative stress are independently associated with cognitive and physical impairment. Thus, these results can help in the early identification and development of strategies for the prevention and treatment of intrinsic capacity decline.

## Introduction

Healthy aging, as described in the World Report on Aging and Health, developed by the World Health Organization, is the process of developing and maintaining intrinsic capacity, which includes all the physical and cognitive functions of an individual (1). Preventing the decline in physical and cognitive abilities is important for maintaining independence and promoting social participation. Further, researchers have indicated that the development of cognitive impairment and physical disability share many common pathophysiological mechanisms (2), and it is important to explore the common pathophysiological mechanisms and biomarkers of both. These results provide an attractive target for evaluating and developing intervention strategies of intrinsic capacity.

Oxidative stress is defined as an imbalance between the release of pro-oxidants and antioxidant defenses in favor of pro-oxidants, which can damage cell structures, leading to health problems. Oxidative stress has been postulated to be an underlying mechanism for the loss of muscle strength. From a biological perspective, with advancing age, the ability of muscle cells to process increased accumulation of reactive oxygen and nitrogen species (ROS/RNS) is compromised, leading to increased oxidative stress responses that impair cellular homeostasis and ultimately the loss of muscle strength and functional decline (3). Previous clinical studies have shown that increased levels of oxidative stress markers are associated with decreased muscle strength (4), sarcopenia (5), and frailty (6). In addition to muscle strength, oxidative stress plays an important role in the progression of Alzheimer's disease (AD) (7). Although the etiology and pathogenesis of AD are not fully understood, oxidative damage is a crucial component of AD. The brain is highly susceptible to oxidative imbalance due to its high energy demand, high oxygen consumption, and relative deficiency of antioxidant enzymes, which directly affect the synaptic activity and neurotransmission in neurons, leading to cognitive impairment (8). In patients with AD and mild cognitive impairment, researchers have found elevated oxidative stress markers in brain tissue or peripheral blood and decreased total antioxidant capacity (9–11). Therefore, the role of oxidative stress in the development of physical and cognitive impairment should be analyzed.

Measurement of ROS is difficult because their half-lives are usually very short; therefore, the evaluation of oxidative stress in biological fluids is conducted by quantifying oxidative stress biomarkers. Previous studies have shown that oxidative stress can damage nucleic acids, lipids, and proteins. In addition, DNA and RNA precursor nucleotides are equally damaged by oxidation. In this regard, 8-oxo-7, 8-dihydro-2′-deoxyguanosine (8-oxodGsn) and 8-oxo-7, 8-dihydroguanosine (8-oxoGsn) are the two main nucleic acid oxidative adducts derived from DNA and RNA, respectively (12). These lesions are caused by oxidative damage at the guanine C-8 position, which, if left unrepaired, can result in G-to-T transversion events (13). However, among these, the most recurring is 8-oxoGsn. Previous studies have shown that RNA is more susceptible to oxidative damage than DNA because of the following reasons: (1) RNA is a single-stranded nucleic acid and is easily exposed to ROS; (2) RNA is less protected by proteins than DNA; (3) the active repair mechanism of oxidized RNA has not been identified; and (4) cytoplasmic RNAs are near the mitochondria, where many ROS are produced. Indeed, oxidative damage to RNA is more common than oxidative damage to DNA in humans (14, 15). Regarding this, 8-oxoGsn is the most detectable and representative RNA oxidative damage product in urine and an indicator of oxidative stress level *in vivo*.

Although the development of muscle strength decline and dementia share common mechanisms, previous studies on oxidative stress and cognitive and muscle strength have usually been studied independently. In addition, blood tests were commonly used as indicators of oxidative stress. Urine samples are easier to obtain, and urinary 8-oxoGsn is stable and easy to store. However, its relationship with physical and cognitive decline in population-based studies has not yet been determined. In this study, we selected commonly used objective indicators of physical function, namely, grip strength, walking speed, and the repeated chair stand test, as well as the Montreal Cognitive Assessment 5-min protocol (MoCA 5-min protocol) as a cognitive assessment tool, and aimed to examine the cross-sectional associations of urinary 8-oxoGsn and physical and cognitive decline to provide a basis for evaluating intrinsic capacity and targeted interventions from a pathophysiological perspective.

## Materials and Methods

### Participants

All participants were recruited from the first wave of the China Aging Longitudinal Study (CALS), an ongoing cohort study that aims to explore health and aging trends in China. Beijing is one of the sub-centers of the CALS program. This study included community-dwelling adults who were conveniently sampled from the population of Beijing. The participants were recruited through advertisements in health examination centers and communities between December 2019 and December 2020. The eligibility criteria were as follows: (1) age >40 years and signed informed consent form; (2) no mental disorders; and (3) no history of alcohol or drug abuse. The exclusion criteria included acute medical treatment or hospitalization within the first 3 months of measurement; the presence of severe diseases, including acute heart, liver, or kidney disease, and respiratory failure; inability to walk independently; and previously diagnosed dementia.

#### Sociodemographic Variables

Trained personnel recorded demographic data during face-to-face interviews. Health characteristics included body mass index (BMI), chronic diseases, and lifestyle. Height and body mass were measured using a digital scale. BMI (kg/m^2^) was calculated as body mass divided by height squared. Chronic diseases included hypertension, diabetes, cerebro-cardiovascular disease, and chronic kidney disease. Smoking habits were categorized as current or never/former smoker, and alcohol consumption was categorized as current or never/former drinker.

#### Physical Performance

Participants were asked to safely complete the grip strength, walking speed, and repeated chair stand tests while the examiners recorded the results. (1) Grip strength was measured using a grip dynamometer (EH101; Camry, Zhongshan, China). During the measurement, the participants stood upright, with their feet naturally separated and their arms relaxed, with full elbow extension. They were instructed to grip the handle as hard as possible. Further, the maximum values obtained from the two tests using the dominant hand were included in the analyses. (2) Walking speed: The participants were asked to walk at their usual pace along a 6-meter course. Additionally, although they were allowed to use a walking aid (a cane, walker, or other walking aid) if necessary, no assistance was provided by another person. The time required to complete the entire distance was recorded in seconds. (3) Repeated chair stand test: The participants were asked to stand from a sitting position without using their arms and to sit down again five times, as quickly as possible, with their arms folded across their chest. The time taken for rising from the chair to standing at the end of the fifth stand was taken and recorded. Cut-offs for maximal grip strength (<28 kg for men and <18 kg for women), walking speed (<1.0 m/s), and repeated chair stand test (≥12 s) were based on the Asian Working Group for Sarcopenia (AWGS) 2019 guidelines (16).

#### Cognitive Function

The MoCA has been recommended as a clinical screening instrument for cognitive impairment; however, it is a relatively long test (~15 mins). In 2005, the National Institute of Neurological Disorders and Stroke-Canadian Stroke Network Harmonization Working Group recommended a very brief protocol (~5 mins), which extracted four subtests from the MoCA (17). The MoCA 5-min protocol was designed to serve as a tool (for bedside screening and busy clinics) that can be used over the telephone and to support large epidemiological and clinical studies.

The MoCA 5-min protocol, which comprises four subtests, assesses five different cognitive domains as follows: attention, verbal learning, memory, executive functions/language, and orientation. In this study, the MoCA 5-min protocol was derived from the Hong Kong version of the MoCA (18). First, simple auditory attention span (also known as immediate memory) was measured using a five-word-learning trial, and the number of words recalled was recorded. Second, the participants were asked to recall as many names of animals as possible in 1 min. While phonemic fluency was proposed in the English version, the Chinese language is based on a non-alphabetic system; therefore, a semantic fluency task (names of animals) was adopted in the study. Third, different weights were given to free and cued recalls, which reflect different types of memory failure (encoding vs. retrieval deficits). The total score of the MoCA 5-min protocol is 30 points; a raw score of <25 in welleducated persons (education >12 years) or a raw score of <23 in less-educated persons (education ≤12 years) was defined as impaired global cognition (19). Table 1 provides a description of the MoCA 5-min protocol. All participants underwent a face-to-face cognitive assessment administered by trained interviewers.

**Table 1:** Description of MoCA 5-min protocol.

**Cognitive domain**	**Item**	**Description**	**Scoring**	**Maximum score**
Attention	1	Immediate recall of 5 words	1 point for each word correctly recalled in the first trial	5
Executive function/language	2	1-min verbal fluency (animal category)	0.5 point for each correct output	9
Orientation	3	6-item date and geographic orientation	1 point for each correct response	6
Memory	4	Delayed recall and recognition of 5 words learned in item 1	2 points for each of the words spontaneously recalled; 1 point for each word by cued recall or recognition but not spontaneously recalled	10
			Total	30

#### Oxidative Stress Biomarker

To prepare the urine samples, the frozen urine was thawed, incubated at 37°C for 5 mins, and centrifuged at 7,500 × g for 5 mins at 4°C. To each 200-μL aliquot of the supernatant, 200 μL of working solution (70% methanol, 30% water with 0.1% formic acid, and 5-mmol/L ammonium acetate) and 10 μL [^15^${\text{N}}_{2}^{13}$C_1_] of 8-oxoGsn were used as an internal standard (240 ρg/L). The mixture was incubated at 37°C for 10 mins and subsequently centrifuged at 12,000 × g for 15 mins at 4°C. Finally, 5 μL of the supernatant was injected for modified ultraperformance liquid chromatography and mass spectrometry analysis. Considering the variability in urinary volumes and significant differences in the renal glomerular function, the concentrations of each of the analytes were normalized relative to the amount of creatinine, and 8-oxoGsn was expressed in μmol/mol.

### Statistical Methods

Urinary 8-oxoGsn was recorded using the 33rd and 66th percentiles as reference. Participants were categorized according to the tertiles of urinary 8-oxoGsn into the lowest, middle, and highest tertile groups. The chi-square statistic was used to test the association of urinary 8-oxoGsn with sex, chronic diseases (hypertension, diabetes, cerebro-cardiovascular disease, and chronic kidney disease), smoking habits, and alcohol consumption. Analysis of variance was used to examine the association of urinary 8-oxoGsn tertiles with selected continuous variables. The relationships between the variables were calculated using Pearson's product-moment correlations.

The following possible confounding factors were considered regarding the association between oxidative stress and physical and cognitive functions: (1) variables that were significantly different among the urinary 8-oxoGsn tertile groups and previously reported to be related to physical and cognitive functions (age, sex, smoking habits, alcohol consumption, hypertension, diabetes, and cerebro-cardiovascular disease), and (2) variables that had no significant difference among the diagnostic groups in this study but were reported to be related to physical and cognitive functions (chronic kidney disease), which were adjusted as covariates.

Multiple linear regression analysis was used to assess the association of physical performance and cognitive function (dependent variables) with tertiles of urinary 8-oxoGsn (independent variables) after adjusting for confounders. The lowest tertile was used as a reference. Logistic regression analysis was performed to examine the correlation between impaired physical and cognitive function (dependent variables) and tertiles of urinary 8-oxoGsn (independent variables) after adjusting for confounders. All analyses were performed using SPSS software (version 25.0; SPSS, Inc, Chicago, IL). Statistical significance was set at *p* < 0.05.

## Results

We studied 1,352 healthy participants (471 men [34.8%] and 881 women [65.2%]). The mean age of participants was 62.9 years (range: 40–94 years). Table 2 shows the health characteristics and cognitive and physical function parameters of the participants according to the tertiles of urinary 8-oxoGsn. Participants in the highest tertile of urinary 8-oxoGsn were, on average, older than those in the middle- and lowest tertiles. There was no significant difference in BMI among the three groups. High prevalence rates of hypertension, diabetes, and cerebro-cardiovascular disease were observed in the highest tertile of urinary 8-oxoGsn. Smoking and alcohol consumption habits were significantly different among the three groups. Regarding physical performance, participants in the highest urinary 8-oxoGsn tertile had a lower grip strength, slower walking speed, and required more time for completing the repeated chair stand test than those in the middle- and lowest tertile groups. In terms of cognitive function, the scores on the MoCA 5-min protocol were significantly lower in the highest tertile group than in the other two groups.

**Table 2:** Sociodemographic characteristics and cognitive and physical function parameters of the participants, according to tertile of urinary 8-oxoGsn.

**Characteristics**	**Urinary 8-oxoGsn**			
	**Lowest tertile, *n* = 445**	**Middle tertile, *n* = 448**	**Highest tertile, *n* = 459**	***P***
Urinary 8-oxoGsn (μmol/mol)	1.0 (0.2)	1.6 (0.2)	2.8 (0.6)	
Age (years)	56.2 (8.6)	61.4 (10.5)	70.6 (11.6)	<0.001
Females	284 (63.8)	284 (63.4)	313 (68.2)	<0.001
Education (years)	14.2 (2.8)	13.7 (3.0)	13.2 (2.9)	<0.001
Health characteristics				
BMI (kg/m^2^)	24.4 (3.1)	24.6 (3.7)	24.5 (3.5)	0.757
Hypertension	128 (28.8)	169 (37.7)	210 (45.7)	<0.001
Diabetes	46 (10.3)	78 (17.4)	102 (22.2)	<0.001
Cerebro-cardiovascular disease	101 (11.0)	110 (16.0)	162 (20.5)	<0.001
Chronic kidney disease	12 (3.1)	15 (3.8)	20 (4.9)	0.387
**Lifestyles**				
Smoking habit	67 (15.1)	49 (10.9)	80 (17.4)	0.019
Alcohol consumption	89 (20.0)	80 (17.8)	63 (13.7)	0.039
**Physical performance**				
Grip strength (kg)	33.0 (8.8)	30.8 (8.6)	25.1 (7.7)	<0.001
Walking speed (m/s)	1.2 (0.2)	1.2 (0.2)	1.0 (0.3)	<0.001
Repeated chair stands test (s)	7.0 (1.8)	7.6 (2.1)	10.9 (2.2)	<0.001
**Cognitive function**				
MoCA 5-min protocol, points	28.5 (2.1)	27.0 (2.4)	26.2 (3.1)	<0.001

*Variables are expressed as mean (SD) or n (%) unless stated. 8-oxoGsn, 8-oxo-7, 8-dihydroguanosine; BMI, body mass index; MoCA, Montreal cognitive assessment*.

Pearson correlation coefficients were used to examine the relationship of urinary 8-oxoGsn levels with physical performance and cognitive function parameters. Significant negative associations were found between the urinary 8-oxoGsn level and grip strength, walking speed, and the MoCA 5-min protocol score (*r* = −0.41, −0.29, and −0.21, respectively; *p* < 0.001), whereas the urinary 8-oxoGsn level was positively associated with the results of the repeated chair stand test (*r* = 0.35, *p* < 0.001). The levels of urinary 8-oxoGsn in participants with different physical and cognitive functional statuses showed significant differences. The urinary 8-oxoGsn levels were significantly higher in the participants with low grip strength (men: <28 kg, women: <18 kg), slow walking speed (<1 m/s), slowly repeated chair stand test (≥12 secs), and impaired cognitive function (MoCA 5-min protocol: <25) than in those with normal values for these parameters (2.72 ± 0.87 vs. 1.73 ± 0.76 μmol/mol; 2.30 ± 0.86 vs. 1.68 ± 0.73 μmol/mol; 2.79 ± 0.78 vs. 1.70 ± 0.74 μmol/mol; 1.95 ± 0.84 vs. 1.63 ± 0.73 μmol/mol, *p* < 0.05). Therefore, higher urinary 8-oxoGsn levels were found to be associated with poor physical performance and worse cognitive function.

**Figure 1:**
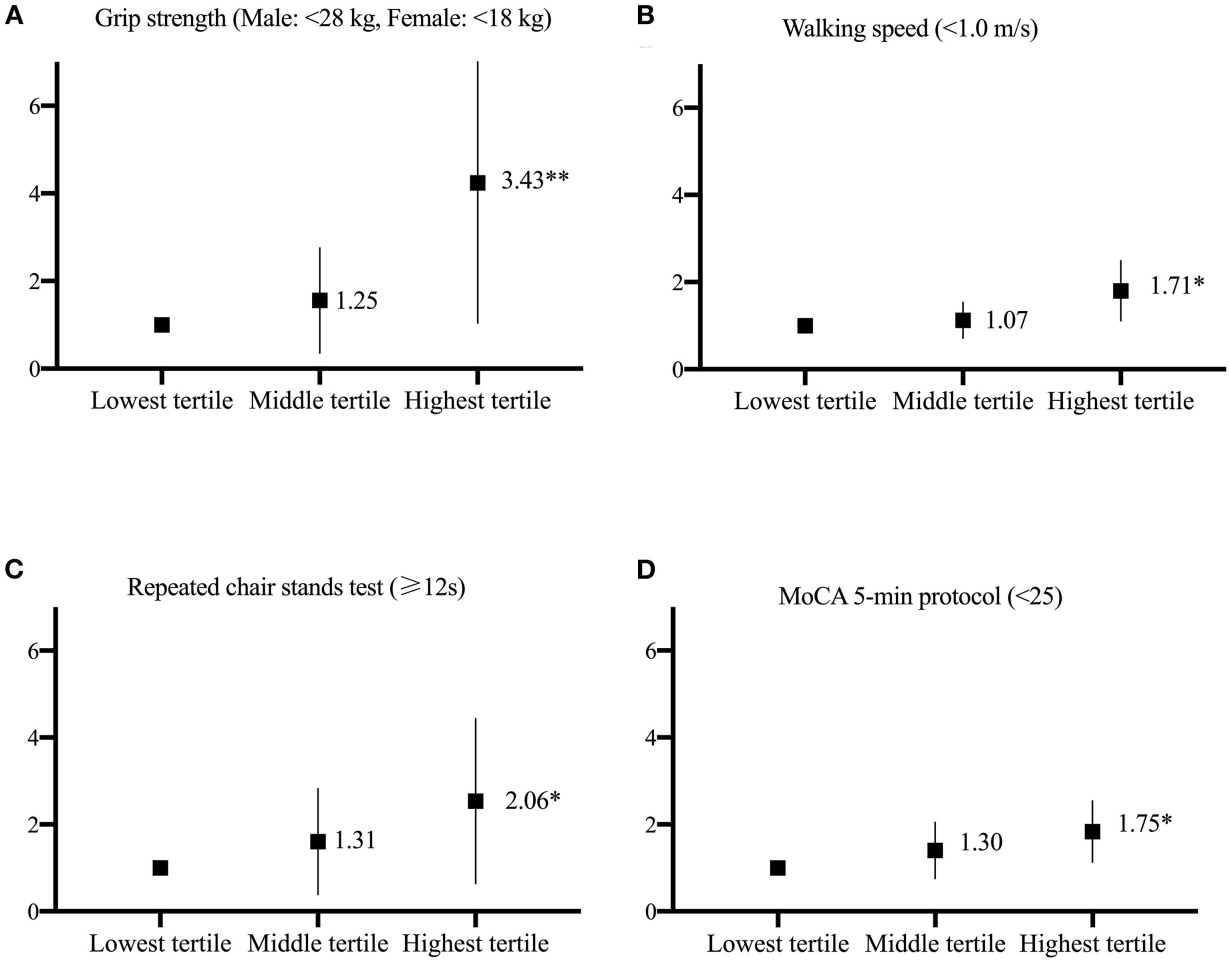
The relationship between the different tertiles of urinary 8-oxoGsn and physical and cognitive function is represented **(A–D)**. Odds ratio and 95% CI derived from logistic regression models after adjusting for age, sex, smoking habits, alcohol consumption, hypertension, diabetes, cerebrocardiovascular disease, and chronic kidney disease. The lowest tertile of urinary 8-oxoGsn was used as the reference group *p < 0.05, **p < 0.001.

After adjusting for confounders, the multiple linear regression method revealed that compared with the participants in the lowest tertile of urinary 8-oxoGsn, those in the highest tertile of urinary 8-oxoGsn had an average decrease in grip strength of 2.55 kg (beta:−2.55, 95% confidence interval [CI]:−3.45 –−1.65), reduction in walking speed of 0.08 secs (beta:−0.08, 95% CI:−0.12 to −0.05), prolonged time of 2.03 sec in the repeated chair stand test (beta: 2.03, 95% CI: 0.97–3.03), and reduction in score on the MoCA 5-min protocol by 0.4 (beta:−0.40, 95% CI:−0.86 to −0.07). In the logistic regression model adjusted for confounders, the participants in the highest tertile of urinary 8-oxoGsn were 3.43 times more likely to have low grip strength (men: <28 kg, women: <18 kg) than those in the lowest tertile (OR 3.43, 95% CI: 1.52–7.76). The participants in the highest tertile of urinary 8-oxoGsn showed a significantly higher risk of impairment in walking speed (<1 m/s) (OR 1.71, 95% CI: 1.16–2.53) and poor performance in the repeated chair stand test (≥12 secs) (OR 2.06, 95% CI: 0.92–4.63) than those in the lowest tertile. In addition, the participants in the highest tertile of urinary 8-oxoGsn were 1.75 times more likely to have cognitive impairment (MoCA 5-min protocol <25) than those in the lowest tertile (OR 1.75, 95% CI: 1.18–2.58). The relationship between the different tertiles of urinary 8-oxoGsn and physical and cognitive impairment is shown in Figure 1.

Previous studies have shown that 8-oxoGsn and physical and cognitive function are age-related. We plotted bar graphs showing the parameters of physical and cognitive function in the three 8-oxoGsn groups in different age categories (Supplementary Materials), further indicating that the relationship between 8-oxoGsn and physical and cognitive function is age-independent.

## Discussion

Our cross-sectional study, which involved more than 1,000 community-dwelling middle-aged or old-aged adults, revealed that increased urinary 8-oxoGsn levels are independently associated with poor physical performance and cognitive impairment; and that a high level of oxidative stress is related to cognitive and physical decline.

### Oxidative Stress and Physical Performance

Decline in muscle function is caused by a decrease in muscle mass and quality. Oxidative stress is supposedly a common determinant of the loss of both muscle mass and quality. This can be explained by the following mechanisms: oxidative stress has the potential to reduce muscle mass by increasing proteolysis and decreasing muscle protein synthesis (20). The mechanisms involved in oxidative stress-mediated muscle quality impairment include reduction in acetylcholine release at the synaptic cleft, leading to a failure of the sarcolemma to produce action potentials (21). This alters the morphology of the motor neuron and neuromuscular junction and ultimately resulting in loss of innervation and reduced number of fibers (21); excitation-contraction coupling failure stemming from redox-induced disruption of calcium release from the sarcoplasmic reticulum (22); and redox modifications to actin and myosin structures that reduce the cross-bridge cycling within the myofibrillar apparatus (23).

Sarcopenia is a clinical sign characterized by diminished skeletal muscle mass and poor muscle function, resulting in frailty and disability (24). Previous studies have shown that elevated oxidative stress is closely linked to age-associated sarcopenia (4). A systematic review showed that frailty and pre-frailty are associated with higher peripheral levels of oxidative stress biomarkers and lower antioxidant parameters (25). A cross-sectional study of 672 women aged ≥65 years reported an independent association between oxidative protein damage and low grip strength, suggesting that oxidative stress might contribute to the loss of muscle strength (26). Oxidative stress has been implicated in sarcopenia and loss of muscle strength with aging; however, the relationship between oxidative stress and decrease in multiple indicators of physical performance has not been sufficiently researched. Our previous research showed that urinary 8-oxoGsn, a recognized biomarker of RNA oxidation, was independently associated with frailty in elderly patients with cardiovascular disease and community-dwelling older adults (27, 28). However, there are limited studies on the correlation between this biomarker and indicators of physical function. This study showed that urinary 8-oxoGsn levels were negatively associated with gait speed and grip strength and positively correlated with the repeated chair stand test results. Based on previous literature, age, sex, lifestyles, and comorbidities, which were considered to have effects on physical function and oxidative stress, were included as confounders (6). After adjusting for potential confounders, high urinary 8-oxoGsn levels were independently associated with poor physical performance.

### Oxidative Stress and Cognitive Function

Although amyloid-b plaques and neurofibrillary tangles have been considered the main cause of AD pathogenesis until now, many studies have proposed that oxidative damage is a key component. The brain is highly susceptible to oxidative damage because of the following four reasons: (1) the brain has a high rate of oxygen consumption; (2) the brain contains high levels of phospholipids and polyunsaturated fatty acids (29); (3) the high levels of transition metals (such as iron and copper) in the brain can catalyze the continual production of free radicals (30); and (4) the brain has low levels of antioxidant enzymes compared with other organs (31). When the level of ROS in the cell exceeds the defense ability of the cell, it undergoes various changes, including modifications of proteins, damage to lipids and DNA, and altered intrinsic properties of biomolecules, which may ultimately lead to cell death. Thus, oxidative stress-mediated biomolecular damage is extensively reported in AD, which suggests the critical role that oxidative stress plays in the development of cognitive impairment.

In 1999, Professor Akihiko Nunomura examined the concentration of 8-oxodGsn/8-oxoGsn in the brain tissue of patients with AD and found that oxidized nucleosides are not mainly distributed in the mitochondrial DNA with high oxidation levels but predominantly occur in the cytoplasmic RNA. This demonstrates that RNA is a major site of nucleic acid oxidative damage and that RNA oxidation is a prominent feature of vulnerable neurons in AD (32). In 2003, Abe et al. found a significant five-fold increase in 8-oxoGsn concentrations in the cerebrospinal fluid of patients with AD compared with healthy controls and that 8-oxoGsn concentrations in the cerebrospinal fluid decreased significantly with the duration of disease and progression of cognitive dysfunction (33). A previous study showed increased RNA oxidative damage in the brains of patients with mild cognitive impairment (34). The results of these *in vivo* studies indicate that an increase in 8-oxoGsn and oxidative RNA damage may play a role in the early stages of AD development. Studies on the relationship between oxidative damage and pathological changes in AD revealed that RNA oxidative damage precedes the formation of amyloid-β or tau pathology (35).

In our previous studies, we found increased amounts of 8-oxoGsn in the hippocampi of senescence-accelerated SAMP8 mice, which exhibited early aging syndromes and declining learning and memory compared with age-matched control mice (36). Furthermore, increased amounts of 8-oxoGsn in RNA were also observed in the hippocampi of patients with AD (36). Notably, we found that increased 8-oxoGsn levels in the RNA of mammals induce the aggregation of amyloid β peptides in cells expressing amyloid precursor protein (37). In previous studies, we revealed that both the 8-oxoGsn content in the brain RNA and urine increased more rapidly in elderly mice, rats, and monkeys, indicating that urinary 8-oxoGsn is a representative marker of oxidative RNA lesions and a biomarker for the sensitive measurement of oxidative stress and aging (37). In this study, we explored the significance of urinary 8-oxoGsn in individuals with undiagnosed dementia with cognitive decline, a relationship that has received minimal attention so far. Results showed that urinary 8-oxoGsn levels were negatively associated with the scores on the MoCA 5-min protocol. Mild cognitive impairment (MCI) is a clinical transitional status between the cognitively normal condition and AD. We recently found that urinary 8-oxoGsn may be a useful indicator for early screening for MCI in frail patients with cardiovascular disease (38). Therefore, it is a promising indicator for the early identification of cognitive impairment and needs to be explored in the future.

### Limitations

This study had some limitations. First, although the results revealed significant associations between oxidative stress biomarkers and physical and cognitive impairments, the cross-sectional approach limits our ability to make causal inferences. Therefore, future high-quality prospective research is warranted to confirm these findings. Second, we used a single biomarker of oxidative stress, and some other oxidative stress indicators were not included in the comparisons. Third, the urinary biomarker used in this study is still under scientific scrutiny, and the reference value range of this marker has not yet been established. Therefore, the tertile method was applied in this study to define the high, middle, and low levels, which limits the interpretation of the results and their clinical applicability.

### Conclusions

Our results suggested a strong relationship between urinary 8-oxoGsn, a biomarker of oxidative damage to RNA, and physical and cognitive performance. Elevated urinary 8-oxoGsn levels are independently associated with cognitive impairment and physical decline. Furthermore, urine samples are easy to obtain, and urinary 8-oxoGsn is stable and easy to store, making it a promising indicator for implementation among community-dwelling older adults. Given the common biological mechanism of oxidative stress in physical decline and cognitive impairment, this study provides an attractive target for the early identification of decline in intrinsic abilities using a biomarker. The results of this study have important implications for the prevention of cognitive and physical decline in the elderly and the development of treatment strategies to promote healthy aging. In the future, cohort studies can be used to observe the causal relationship and degree of influence of this urinary indicator on cognitive and physical function decline; this indicator can be used to observe the efficacy of anti-oxidative stress treatment.

## Data Availability Statement

The original contributions presented in the study are included in the article/Supplementary Material, further inquiries can be directed to the corresponding author/s.

## Ethics Statement

The study protocol was approved by the Research Ethics Committee of Beijing Hospital (2019BJYYEC-054-02), and the study was performed in accordance with the principles of the Declaration of Helsinki. The patients/participants provided their written informed consent to participate in this study.

## Author Contributions

J-PC and SJ: conceptualization and design, writing—review & editing, and supervision. SJ: methodology, writing—original draft, and formal analysis. JC, L-QZ, ZL, YZ, and YS: acquisition of subjects and data. J-PC and JC: resources and project administration. All authors contributed to the article and approved the submitted version.

## Funding

This work was supported by the National Key R&D Program of China [2018YFC2000301 and 2020YFC2002700] and CAMS Innovation Fund for Medical Sciences [2021-1-I2M-1-050].

## Conflict of Interest

The authors declare that the research was conducted in the absence of any commercial or financial relationships that could be construed as a potential conflict of interest.

## Publisher's Note

All claims expressed in this article are solely those of the authors and do not necessarily represent those of their affiliated organizations, or those of the publisher, the editors and the reviewers. Any product that may be evaluated in this article, or claim that may be made by its manufacturer, is not guaranteed or endorsed by the publisher.
