# Supplementary figures and images for: Role of a Urinary Biomarker in the Common Mechanism of Physical Performance and Cognitive Function
Source: Front Med (Lausanne). 2022 Feb 18;9:816822. doi: 10.3389/fmed.2022.816822 (PMC8894651; doi:10.3389/fmed.2022.816822)

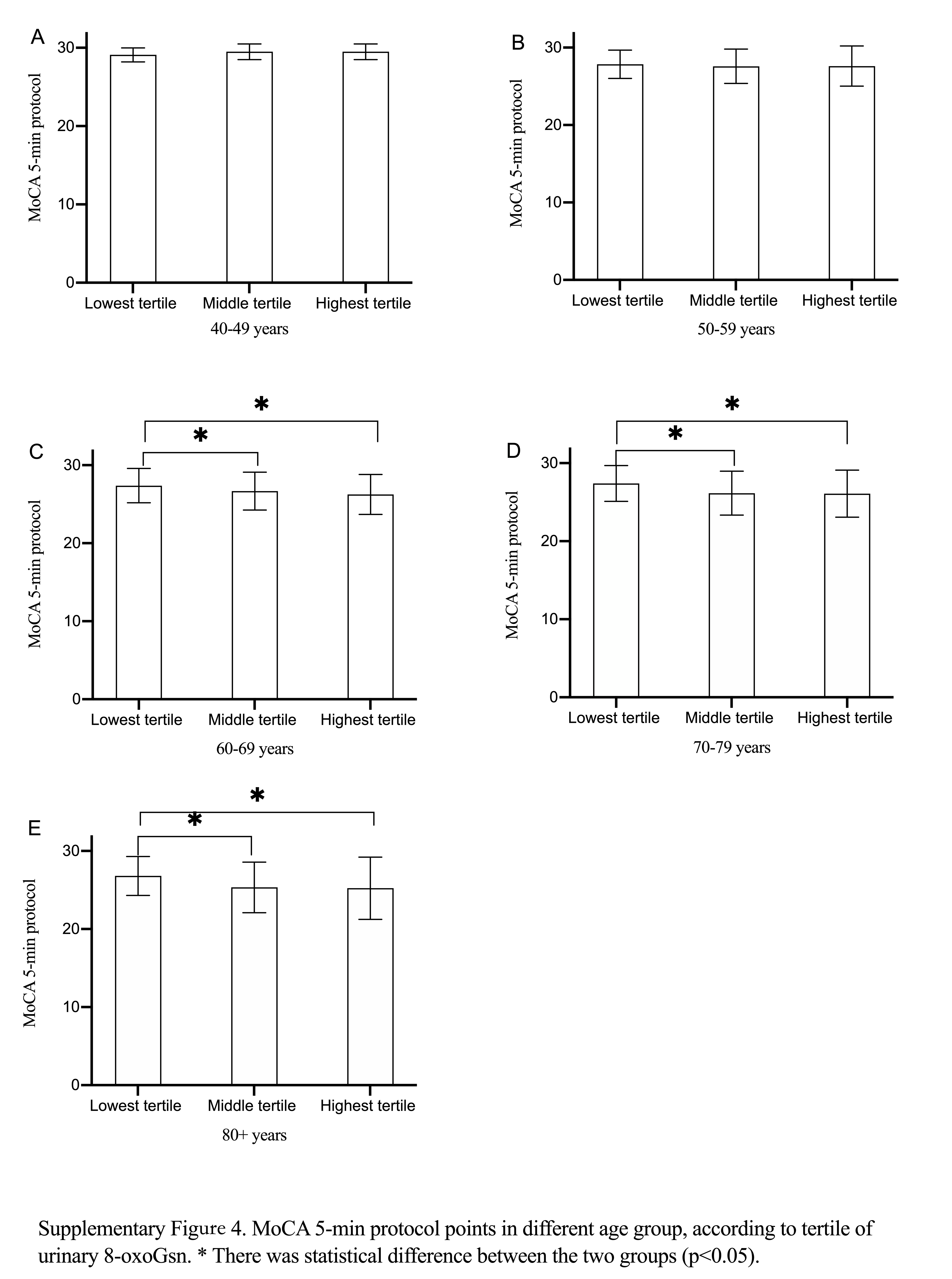

Supplement: Supplementary file 1 [file Data_Sheet_1.ZIP › Supplementary/Fig S4_MoCA 5-min protocol.tiff]

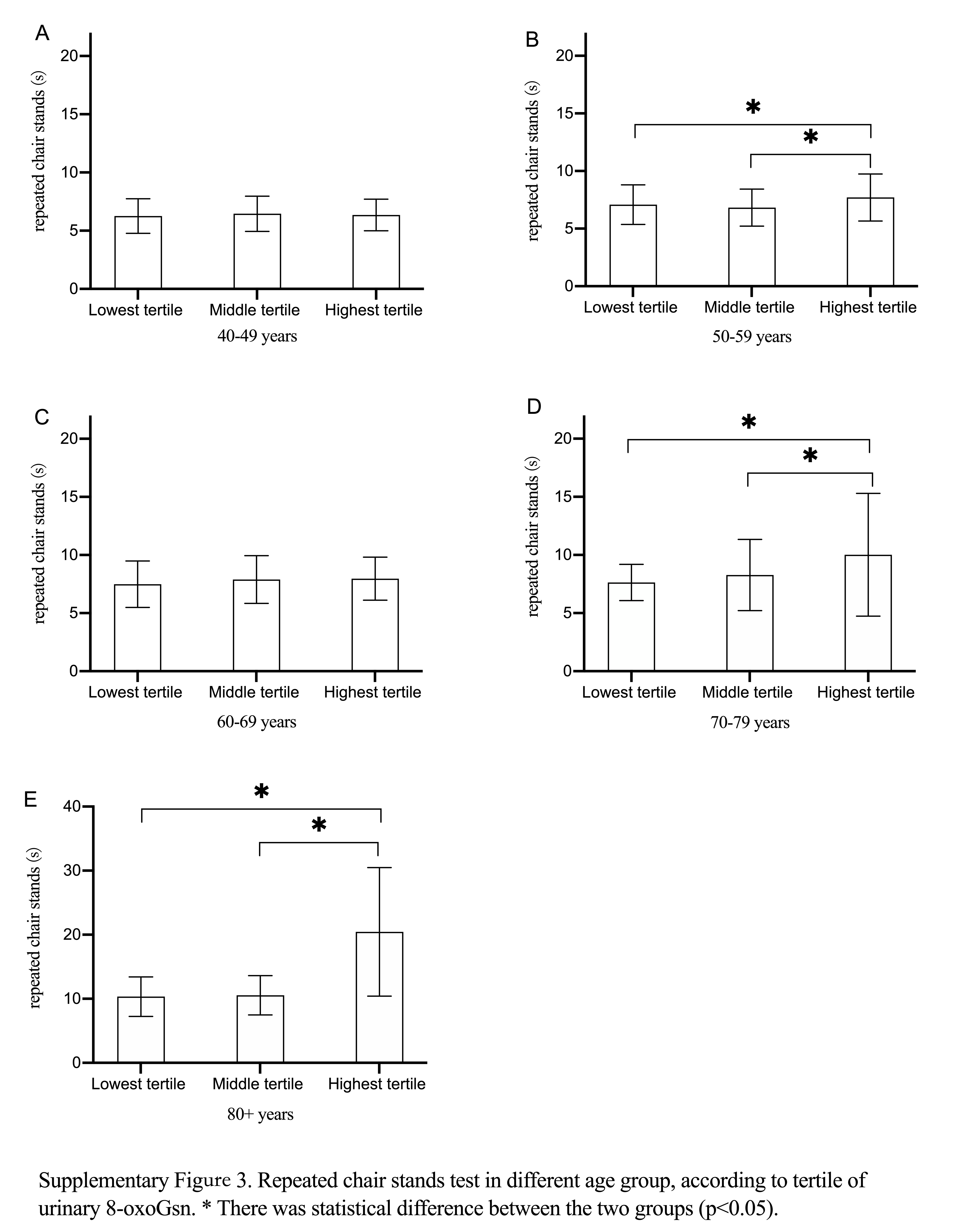

Supplement: Supplementary file 1 [file Data_Sheet_1.ZIP › Supplementary/Fig S3_repeated chair stands test.tiff]

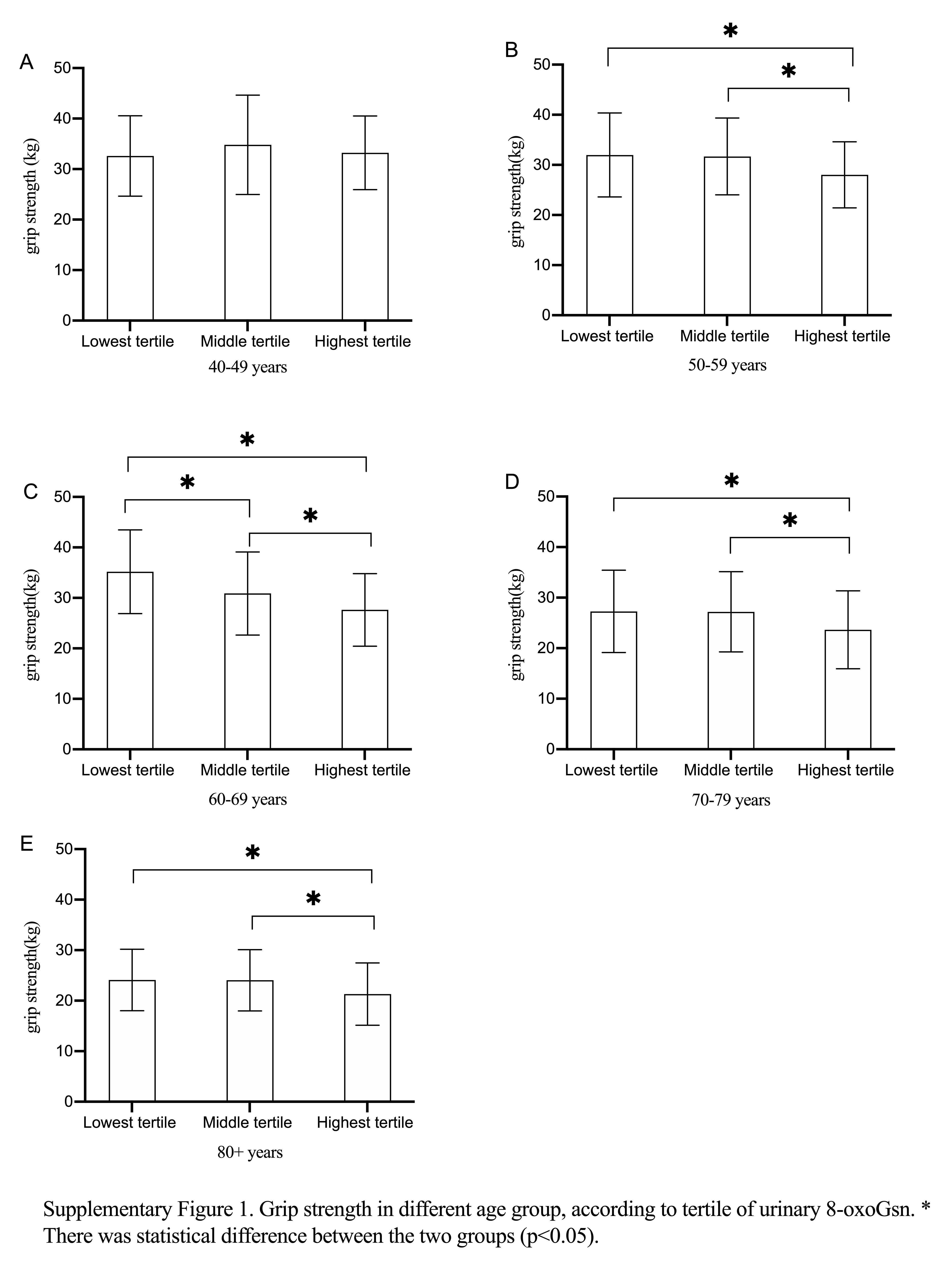

Supplement: Supplementary file 1 [file Data_Sheet_1.ZIP › Supplementary/Fig S1_grip strength.tiff]

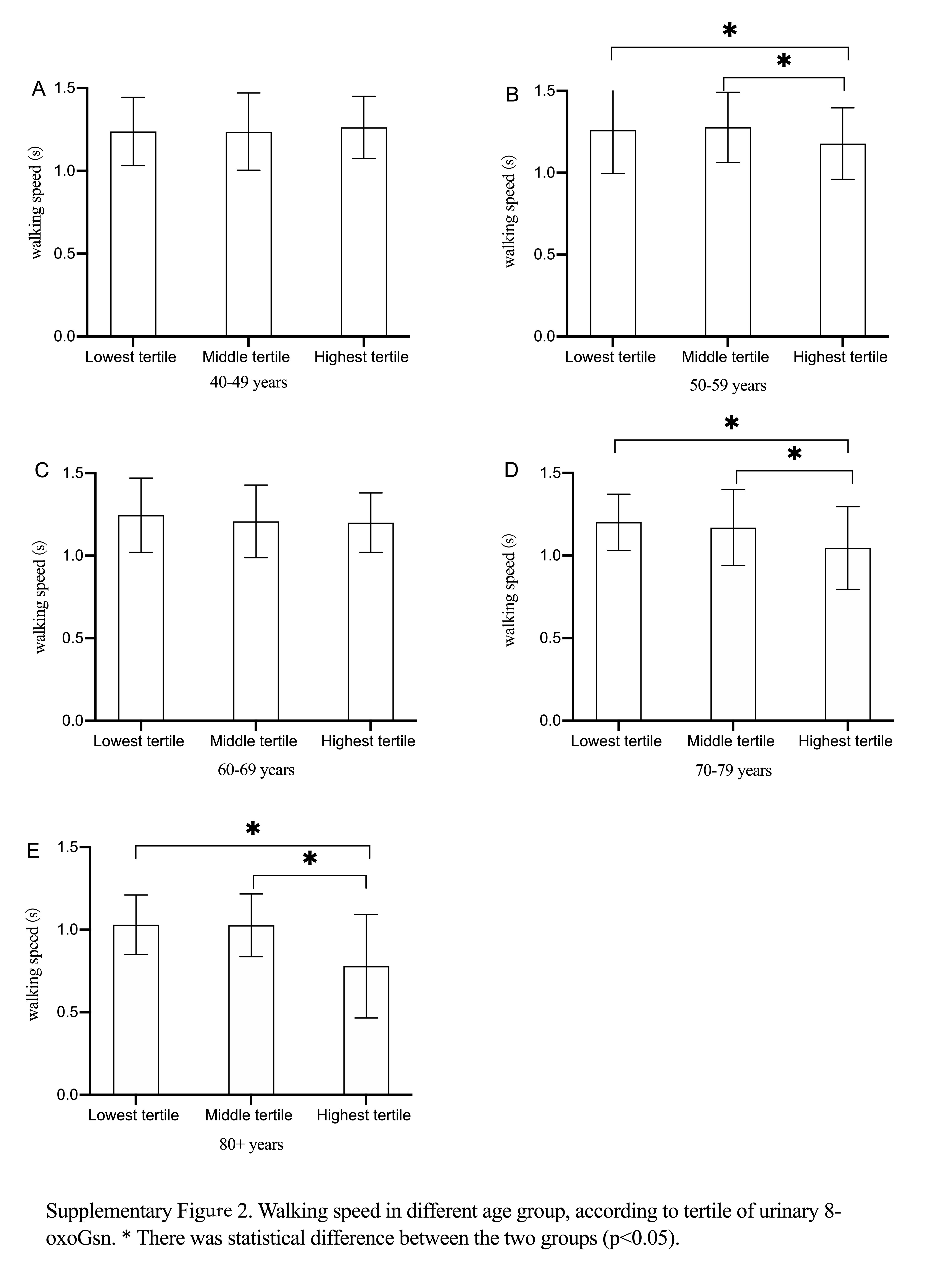

Supplement: Supplementary file 1 [file Data_Sheet_1.ZIP › Supplementary/Fig S2_walking speed.tiff]
